# A single enzyme becomes a Swiss Army knife

**DOI:** 10.1371/journal.pbio.3003072

**Published:** 2025-04-02

**Authors:** Andreas Sichert

**Affiliations:** Institute of Molecular Systems Biology, ETH Zürich, Zurich, Switzerland

## Abstract

An alga that abandoned photosynthesis? This Primer explores a PLOS Biology study showing that a single horizontal gene transfer event allowed the diatom Nitzschia sing1 to evolve a complete enzymatic machinery to break down alginate from brown algae, unlocking a new ecological niche.

## Teaser

An alga that abandoned photosynthesis? A study published in PLOS Biology shows that from a single horizontal gene transfer event, the diatom *Nitzschia sing1* evolved a complete enzymatic machinery to break down alginate from brown algae, unlocking a new ecological niche.An alga that abandoned photosynthesis? This Primer explores a PLOS Biology study showing that a single horizontal gene transfer event allowed the diatom Nitzschia sing1 to evolve a complete enzymatic machinery to break down alginate from brown algae, unlocking a new ecological niche.

Next time you walk along the beach, chances are you will stumble over a fascinating piece of evolution that was recently unraveled in a study published in PLOS Biology [1]. Sometimes you just find bits and pieces of them washed up on the shore, but usually they are hidden from plain sight, growing just below the water’s surface—brown algae. And on their surfaces, you might be able to find the diatom *Nitzschia sing1* who abandoned its original photosynthetic lifestyle, embarking on an evolutionary journey to thrive on algae derived sugars.

Brown algae are not only the photosynthetic cornerstone of many coastal ecosystems but also serve as a major food source for heterotrophic microbes. Similar to cellulose of land plants, alginate is one of the major structural polysaccharides in the cell walls of brown algae. Consequently, research efforts focus on the molecular enzymatic mechanisms that control the recycling of these carbon molecules in the ocean [2] and exploring their potential for converting algal biomass into valuable resources, such as biofuel [3].

To thrive as a heterotroph on algal surfaces, *N. sing1* adapted to break down alginate by evolving the degradation pathway from a single horizontal gene transfer (HGT) event: *N. sing1* acquired an alginate lyase gene from a marine bacterium. This ancestral enzyme was likely able to cleave the alginate polysaccharide into smaller oligosaccharides. However, the breakdown process usually requires further steps, such as transporting these oligosaccharides into the cell and further degrading them into monosaccharides for energy metabolism, that the diatom needed to evolve. Once integrated into the diatom’s genome, the ancestral alginate lyase underwent extensive duplication and diversification, giving rise to 91 enzymes grouped into three distinct gene families, each with specialized roles. Using a combination of biochemical assays, structural modeling, and genomic analyses, the team of researchers demonstrated how sequence diversity within these families directly underpins their unique functions for alginate degradation.

The CA family retained the ancestral role as an endolytic enzyme, cutting the alginate polysaccharide in the middle of the chain producing smaller oligosaccharides. Thus, CA family enzymes serve as the “first scissor” in alginate degradation, initiating the breakdown process by producing large chunks of food. Meanwhile, the A^n^C^n^ family acquired an exolytic mode of action successively cleaving monosaccharides from the end of alginate oligosaccharides. Thus, the A^n^C^n^ proteins serve as “knives”, slicing off edible portions of food from large chunks. This significant functional shift in the A^n^C^n^ family enzymes was driven by a 15-base pair insertion, which restructured the enzymes’ catalytic pockets to favor terminal cleavage rather than internal cutting. This adaptation is critical for providing the diatom with the simpler sugar building blocks needed for energy acquisition. In contrast, members of the A^n-TMD^ family lost their catalytic activity and additionally acquired a transmembrane domain. These proteins appeared to be tethered to the cell surface and potentially have a functional role in the cellular transport of alginate-derived substrates. Thus, the A^n-TMD^ family acts as “bottle opener” enabling controlled nutrient transport. Together, these findings illustrate how gene duplication, followed by neofunctionalization, allowed *N. sing1* to assemble a versatile toolkit capable of fully exploiting the energy-rich polysaccharides found in brown algal cell walls.

The massive expansion of a single enzyme family in *N. sing1* echoes a pattern observed in other highly specialized polysaccharide degrading microorganisms. For example, bacterial populations degrading chitin or alginate evolve through acquiring diverse enzyme repertoires via HGT [4,5]. In these cases, HGT enables rapid adaptation of the enzyme repertoires to efficiently break down differently sized polysaccharide fragments or simply increase overall degradation through gene dosage. Similarly, plant pathogens such as the infamous fungi *Phytophthora infestans* expanded their enzyme repertoires to improve host infection and resource acquisition [6]. However, *N. sing1* takes a slightly different evolutionary route. Rather than relying solely on HGT, it achieves enzyme diversity within a single species through homologous recombination and gene duplication. This distinction highlights how different evolutionary strategies can converge to address similar ecological challenges, whether through interspecies gene sharing in bacteria or internal genomic innovation in diatoms. These examples emphasize the central role of enzyme expansion for adaptation towards specialized polysaccharide metabolism and the diverse mechanisms that underpin it.

The evolution of *N. sing1*’s enzymatic repertoire and its subsequent change of lifestyle reveal how genomic innovation can redefine ecological roles, demonstrating the incredible adaptability of life. By unraveling how this diatom transitioned from photosynthesis to heterotrophy, the present study broadens our understanding of microbial evolution and highlights the interplay between genetics and ecological function. As the authors note, the ability to connect genetic changes with metabolic adaptations provides a foundation for exploring how other microbes may evolve to exploit new ecological niches. The insights gained here may not only inspire biotechnological applications for sustainable biomass processing, such as using algal degradation pathways to upcycle marine waste, but also deepen our appreciation for the evolutionary creativity of microbes. While we wait for the biotechnological aspirations to pan out, we can enjoy that we’ve uncovered the peculiar and remarkable lifestyle of some of the smallest and most fascinating inhabitants of our coastal ecosystems.

## References

[1] Lim Z, Zheng P, Quek C, Nowrousian M, Aachmann FL, Jedd G. Diatom heterotrophy on brown algal polysaccharides emerged through horizontal gene transfer, gene duplication and neofunctionalization. PLOS Biol. 2025;23:e3003038. doi: 10.1371/journal.pbio.3003038 40168346 PMC11960938

[2] Arnosti C, Wietz M, Brinkhoff T, Hehemann J-H, Probandt D, Zeugner L, et al. The biogeochemistry of marine polysaccharides: sources, inventories, and bacterial drivers of the carbohydrate cycle. Annu Rev Mar Sci. 2021;13:81–108. doi: 10.1146/annurev-marine-032320-095045 32726567

[3] Wargacki AJ, Leonard E, Win MN, Regitsky DD, Santos CNS, Kim PB, et al. An engineered microbial platform for direct biofuel production from brown macroalgae. Science. 2012;335:308–13. 22267807 10.1126/science.1214547

[4] Hehemann J-H, Arevalo P, Datta MS, Yu X, Corzett CH, Henschel A, et al. Adaptive radiation by waves of gene transfer leads to fine-scale resource partitioning in marine microbes. Nat Commun. 2016;7:12860. doi: 10.1038/ncomms12860 27653556 PMC5036157

[5] Datta M, Sliwerska E, Gore J, Polz M, Cordero OX. Microbial interactions lead to rapid micro-scale successions on model marine particles. Nat Commun. 2016;7:11965. 27311813 10.1038/ncomms11965 PMC4915023

[6] Sabbadin F, Urresti S, Henrissat B, Avrova AO, Welsh LRJ, Lindley PJ, et al. Secreted pectin monooxygenases drive plant infection by pathogenic oomycetes. Science. 2021;373:774–9. 34385392 10.1126/science.abj1342

